# Real Time Observation of Single Membrane Protein Insertion Events by the *Escherichia coli* Insertase YidC

**DOI:** 10.1371/journal.pone.0059023

**Published:** 2013-03-19

**Authors:** Sophie Winterfeld, Stefan Ernst, Michael Börsch, Uwe Gerken, Andreas Kuhn

**Affiliations:** 1 Institute of Microbiology and Molecular Biology, University of Hohenheim, Stuttgart, Germany; 2 3^rd^ Institute of Physics, University of Stuttgart, Stuttgart, Germany; Centre National de la Recherche Scientifique, Aix-Marseille Université, France

## Abstract

Membrane protein translocation and insertion is a central issue in biology. Here we focus on a minimal system, the membrane insertase YidC of *Escherichia coli* that inserts small proteins into the cytoplasmic membrane. In a reconstituted system individual insertion processes were followed by single-pair fluorescence resonance energy transfer (FRET), with a pair of fluorophores on YidC and the substrate Pf3 coat protein. After addition of N-terminally labeled Pf3 coat protein a close contact to YidC at its cytoplasmic label was observed. This allowed to monitor the translocation of the N-terminal domain of Pf3 coat protein across the membrane in real time. Translocation occurred within milliseconds as the label on the N-terminal domain rapidly approached the fluorophore on the periplasmic domain of YidC at the trans side of the membrane. After the close contact, the two fluorophores separated, reflecting the release of the translocated Pf3 coat protein from YidC into the membrane bilayer. When the Pf3 coat protein was labeled C-terminally, no translocation of the label was observed although efficient binding to the cytoplasmic positions of YidC occurred.

## Introduction

A fundamental question of membrane protein insertion is how the hydrophilic parts of a protein are translocated across the membrane and how its hydrophobic parts are properly oriented and inserted into the lipid bilayer [1], [2]. Membrane insertion of small bacterial, chloroplastic and mitochondrial proteins is catalyzed by members of the Oxa-1 family, like YidC of *E. coli* [2], [3]. In bacteria, YidC is an essential protein and mutations in the YidC homologues of animals and plants cause severe defects in mitochondria [4] and chloroplasts [5]. YidC functions either alone in the so-called YidC-only pathway or in conjunction with the Sec translocase [3]. In contrast to the Sec translocase, YidC consists of a single protein component making it an ideal minimal system for *in vitro* studies. From its sequence it is predicted that YidC has 6 transmembrane segments; the N- and C-terminus as well the C1 loop with 50 residues and the C2 loop with 16 residues, respectively, are in the cytoplasm. The periplasmic loops P1, P2 and P3 consist of 354, 23 and 3 amino acid residues, respectively.

YidC purification and reconstitution into liposomes allowed efficient insertion of the c-subunit of the F_o_F_1_-ATP synthase [6] and of the major coat protein of bacteriophage Pf3 [7]. Biochemical data [8] suggest that the newly synthesized Pf3 coat protein approaches the lipid bilayer in a first binding step. It then interacts with 4 of the 6 transmembrane segments [9]. The hydrophilic N-terminal domain is translocated through the membrane during the insertion process while the hydrophobic region of the substrate is contacting YidC along the entire membrane spans. Although the individual steps of the insertion process are documented by several mutants, the actual process has not been observed. Here we follow the individual steps of membrane insertion by single molecule microscopy. After the addition of Pf3 coat protein to YidC-containing proteoliposomes the Pf3 protein readily binds to YidC and as it traverses the lipid bilayer it colocalized with the YidC insertase. We found that residues located at the cytoplasmic surface and at the periplasmic surface of Pf3 coat protein are in close contact with YidC during membrane insertion. The actual time the Pf3-YidC contact lasted was in the millisecond range. As a last step, the inserted protein separated from YidC for partitioning into the lipid bilayer as the two fluorescent probes lost their close contact.

## Results

### Atto520-labeled Pf3 Coat Proteins and Atto647N-labeled YidC

**Figure 1:**
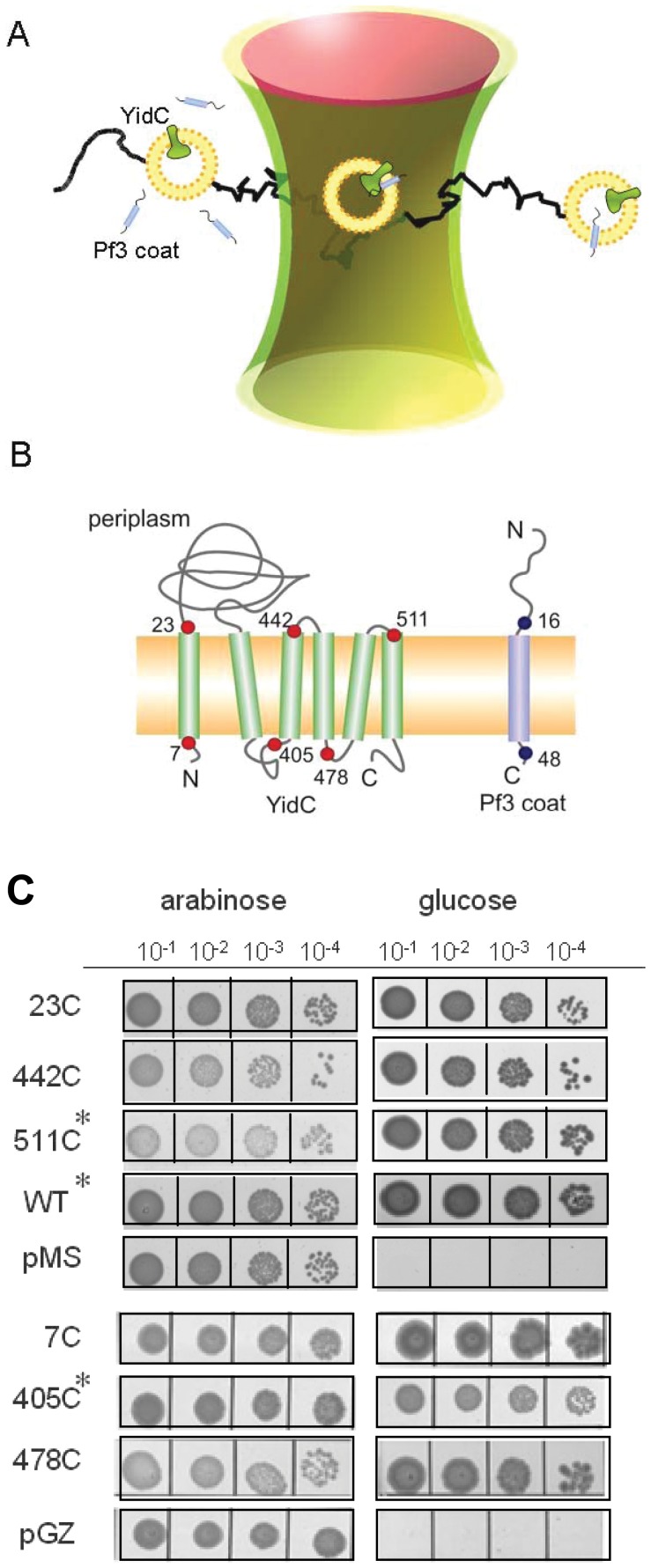
Single-pair FRET experiment of membrane protein insertion in solution. (A) Donor-labeled Pf3 coat protein was added to proteoliposomes containing acceptor-labeled YidC protein and fluorescence was followed in the confocal volume. (B) Positions of the donor fluorophor (Atto520, blue dots) at residues 16 and 48 in the Pf3 coat protein and the acceptor fluorophor (Atto647N, red dots) at periplasmic (23, 442, 511) and cytoplasmic (7, 405, 478) residues of YidC. (C) Complementation assay of the YidC single-cysteine mutants. Growth under glucose conditions indicates functional complementation. Asteriks denote added 1 mM IPTG in the glucose containing LB agar to optimize expression.

To investigate the insertion process in molecular detail, a single-pair fluorescence resonance energy transfer (FRET) approach was developed (Fig. 1A). First, single cysteine mutants of the Pf3 coat protein and of the YidC protein were generated. The mutant Pf3 coat proteins were purified and labeled with the FRET donor fluorophore Atto520-maleimide on cysteines at the amino acid positions 16 or 48, respectively. These positions were chosen to have fluorophores located on the different sides of the membrane, when the protein is inserted. Single cysteine mutants of YidC were purified and labeled with Atto647N-maleimide as FRET acceptor at positions on either side of the membrane, i.e. at the residues 7, 23, 405, 442, 478 or 511, respectively (Fig. 1B). All single cysteine mutants were capable to replace YidC in an *in vivo* complementation assay and are therefore functional (Fig. 1C). This was tested in *E. coli* MK6S where the chromosomal *yidC* gene is under control of the arabinose BAD promoter [10]. Cells were grown overnight in LB medium containing 0.2% (w/v) arabinose and spotted onto LB agar containing 0.2% glucose to deplete YidC. The cells expressing the wild-type YidC or any mutant YidC allowed bacterial growth.

### Confocal FRET Microscopy of Membrane Protein Insertion

Atto647N-YidC was reconstituted in phosphatidyl choline (DOPC) to obtain proteoliposomes. Topological analysis with trypsin showed that more than 80% of YidC was reconstituted in an orientation where the periplasmic P1 domain is in the lumen of the proteoliposomes. We focused two lasers in a common detection volume of 10±1 fL and recorded photon bursts from single proteoliposomes with the fluorescent proteins as they diffused through the confocal volume (Text S1 and S2). A continuous-wave laser with 514 nm was used to excite the Atto520-Pf3 coat protein, and interleaved with short laser pulses at 635 nm to probe the presence Atto647N-YidC in the detection volume.

Previous experiments [11] had shown that Atto520-labeled Pf3 coat protein is readily inserted into proteoliposomes with one defined topology. Whereas an amino-terminal location of the label is translocated and protected from quenching, a carboxy-terminal location of Atto520 is fully quenched. Here, the purified and Atto520-labeled Pf3 coat protein was added to the proteoliposomes present in a buffer droplet onto a cover glass on a confocal microscope and fluorescence bursts were measured for 420 s after the Pf3 protein addition. Most bursts occurred within the first 240 s during which the membrane insertion of most Pf3 proteins is observed [11]. After Atto520-Pf3 coat protein was added, colocalization of both proteins in the same proteoliposome was observed by FRET between donor and acceptor dyes in single photon bursts. These bursts with a constant or a changing FRET efficiency lasted between 14 and 60 ms and were statistically analyzed (Text S1). The final concentrations of both labeled proteins were about 0.2 nM.

**Figure 2:**
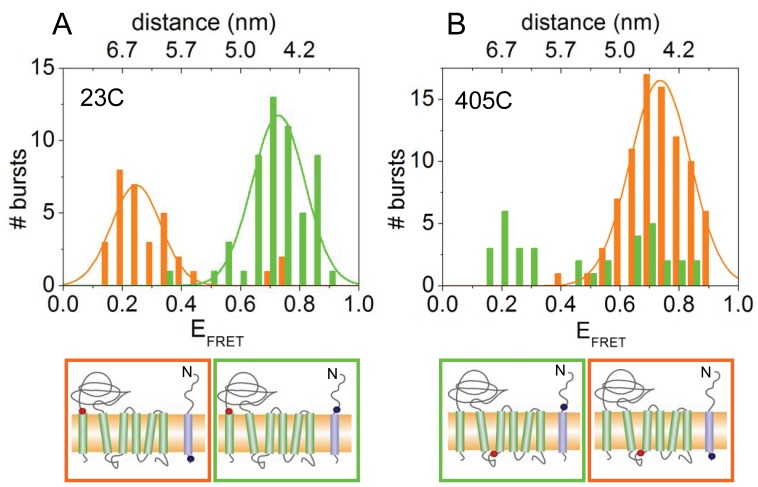
FRET histograms of YidC labeled at position 23 in the periplasmic region (A) and at position 405 in the cytoplasmic region (B). Pf3 coat protein labeled at residue 16C (green bars) or 48C (red bars) was added, respectively. FRET efficiencies of individual bursts of Atto520-Pf3 protein with Atto647N-YidC and the resulting distance between the two probes were calculated. The positions of the Atto520 and Atto647N label are indicated by blue and red dots, respectively (lower panels).

The FRET efficiencies within photon bursts that contained YidC as well as Pf3 coat protein were first analyzed (Fig. 2). Since the FRET efficiency depends on the spatial distance between of the donor-acceptor probes [12], [13] FRET efficiency can be directly translated into the distances of between either the N- or C-terminal regions of Pf3 coat protein and the periplasmic and cytoplasmic regions of YidC.

When the N-terminally-labeled Pf3 protein (Atto520-Pf3-16C) was added, photon bursts showed high FRET efficiencies with the reconstituted YidC protein labeled periplasmically (Atto647N-YidC-23C; Fig. 2A, green bars). This indicates that the fluorescent donor in the N-terminal region of the Pf3 protein is in close proximity with the periplasmically localized acceptor of YidC, and that the Pf3 coat protein has therefore reached a transmembrane configuration. In contrast, the C-terminally-labeled Pf3 coat protein (Atto520-Pf3-48C) showed no such high FRET efficiencies with YidC labeled on the periplasmic side (Fig. 2A, red bars). When Atto520-Pf3-16C was added to YidC labeled at the cytoplasmic side (Atto647N-YidC-405C), photon bursts showing high FRET efficiencies were frequently detected (Fig. 2B, green bars), suggesting that the N-terminal region of Pf3 contacts YidC already before its actual translocation. However, Pf3-48C did show high FRET efficiencies with the cytoplasmically labeled YidC proteins (Fig. 2B, red bars), suggesting that the C-terminal region of bound Pf3 coat protein closely contacts YidC at its cytoplasmic domain during the insertion event.

### Mapping Other Positions of YidC

**Figure 3:**
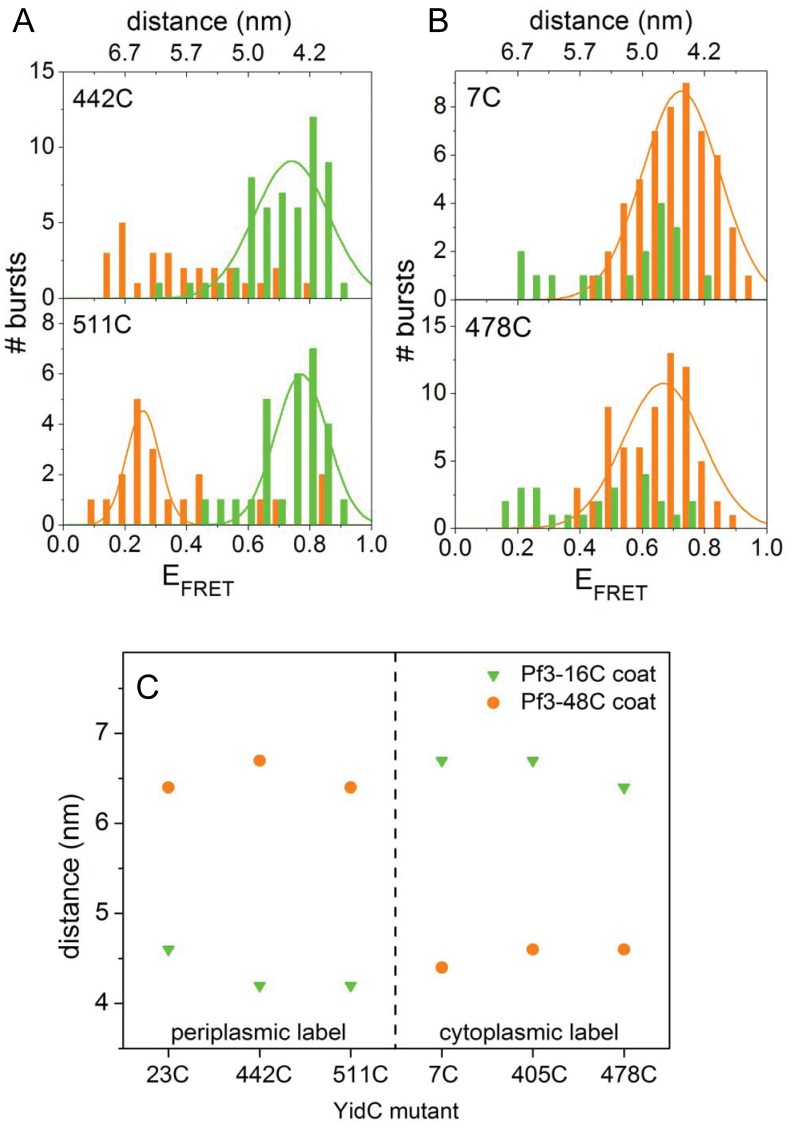
FRET histograms of YidC labeled at positions 442 or 511 in the periplasmic region (A) and at position 7 or 478 in the cytoplasmic region (B). Pf3 coat protein labeled at residue 16C (green bars) or 48C (red bars) was added, respectively. FRET efficiencies of individual bursts of Atto520-Pf3 protein with Atto647N-YidC and the resulting distance between the two probes were calculated. (C) Minimal distances of Atto520-Pf3 protein with Atto-647N-YidC at the periplasmic positions 23C, 442C, and 511C (left part of panel) and the cytoplasmic positions 7C, 405C, 478C (right part of panel). The Pf3 coat protein was labeled at position 16C (green triangles) or at position 48C (red circles), respectivley.

Membrane insertion of Atto520-Pf3 into other Atto647N-YidC containing proteoliposomes confirmed the results we had obtained with YidC-23C and YidC405C, respectively (Fig. 3A). YidC was labeled with Att647N at the periplasmic positions 442 and 511 of loop P2 and P3, respectively, and at the cytoplasmic positions 7 and 478 (Fig. 3B). The periplasmically labeled YidC proteins showed high FRET efficiencies only with Atto520-Pf3-16C (Fig. 3A, green bars), whereas the cytoplasmically labeled YidC were approached by Atto520-Pf3-48C (Fig. 3B, red bars). After the minimal donor-acceptor distances were statistically analyzed for each FRET pair (Fig. 3C) a clear distinction between the transmembrane donor-acceptor pairs and those located at the same side of the membrane can be made. Whereas the latter distances (4.2–4.6 nm) are limited by the Förster radius [12], [13], the transmembrane distances (6.4–6.7 nm) are determined by the width of the DOPC membrane bilayer of about 6 nm [14].

### Time-resolved FRET Analysis of Single Membrane Insertion Events

**Figure 4:**
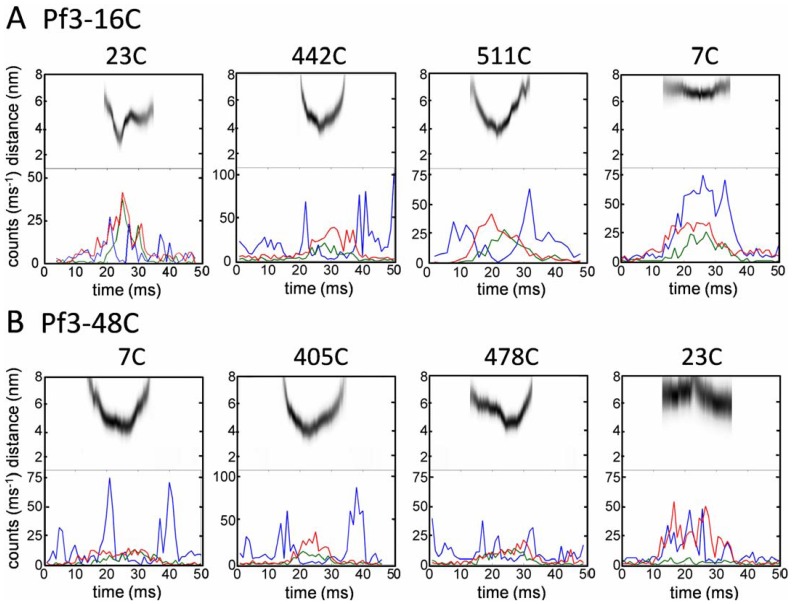
Individual time traces of photon bursts and calculated FRET donor-acceptor distances. Donor fluorescence (blue line) and FRET acceptor (green line) were recorded following 514 nm excitation. The presence of the acceptor was verified with interleaved 635 nm laser pulses (red line). (A) Donor-acceptor contacts were analyzed with Pf3-16C at the periplasmic sites of YidC (23C, 442C, 511C) and one cytoplasmic site (7C) and (B) with Pf3-48C at the cytoplasmic sites of YidC (7C, 405C, 478C) and for a control at one periplasmic site (23C).

Individual recordings can be powerful in providing information on the kinetics of approach and divergence of the two labeled molecules in real time. Therefore, the FRET traces were individually analyzed and the distance of the two fluorophores was followed for 50 ms (Fig. 4). When the N-terminally labeled Pf3-16C approached a periplasmically labeled YidC protein (23C, 442C, 511C) high FRET efficiencies were observed and the calculated distance between the donor and acceptor dyes reached a minimal value of about 3.5 nm (Fig. 4A). This indicates that the close contact between the Pf3 coat protein and YidC lasts for ∼10 ms. The distance between the two fluorophores is clearly shown by the distance traces (gray) in the upper part of each panel. First, the two proteins approached each other and then separated again most likely by lateral diffusion. When the two proteins approached each other the acceptor fluorescence increased (green line) and the donor fluorescence decreased (blue line). When the two fluorophores were located on opposite sides of the membrane (far right panels) minimal distances of only 6 to 7 nm were observed. In cases when a contact at the periplasmic side of YidC was observed translocation of the N-terminal domain of the Pf3 coat protein had to be completed. The actual membrane translocation was assumed to occur immediately before two periplasmic dyes came into their closest contact.

**Figure 5:**
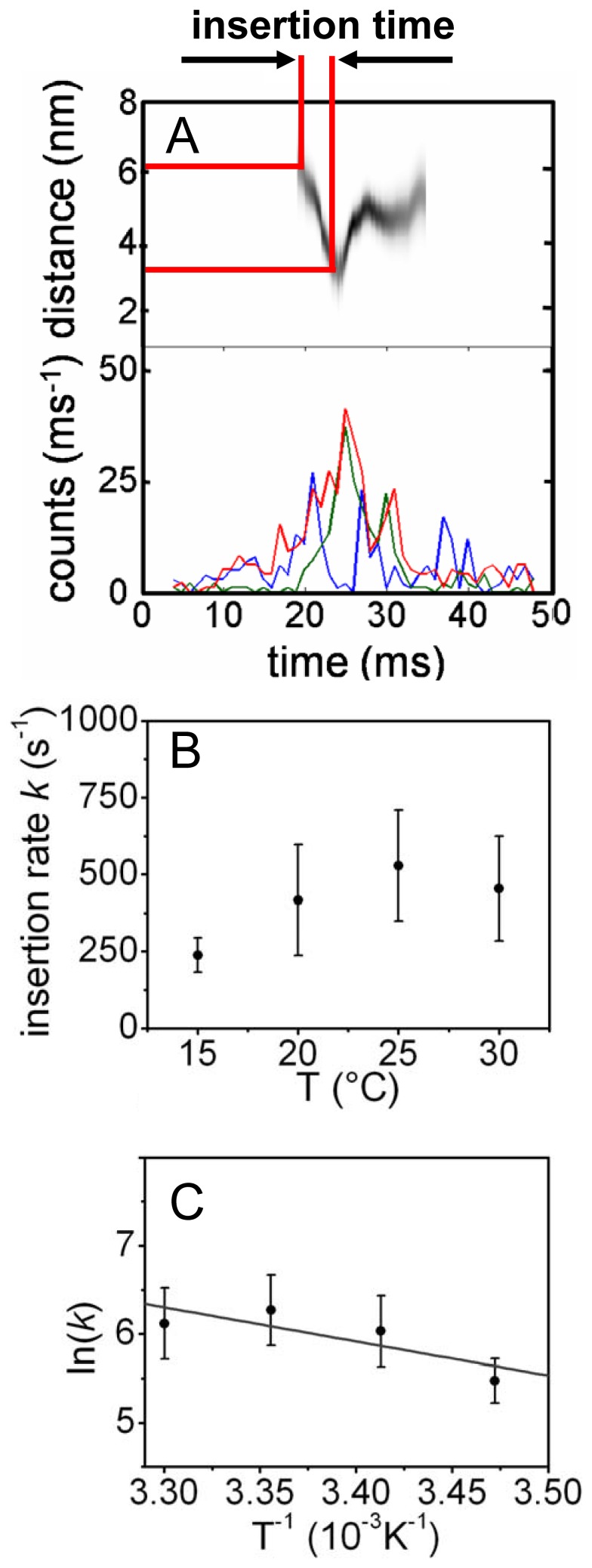
Time-resolved analysis of FRET events. (A, upper panel) Time-dependent distances between Pf3-16C coat and YidC-23C during insertion process, and (A, lower panel) the corresponding FRET event. Blue trace: Atto520 labeled Pf3 coat; green trace: Atto647N labeled YidC; red trace: acceptor test signal. The definition of the insertion time is graphically shown in the upper panel. (B) Temperature dependence of the insertion rate *k* and (C) the corresponding Arrhenius-plot.

Since the insertion of Pf3 coat protein is catalyzed by YidC [7] we expected a temperature-dependence of the time and insertion rate, respectively. For YidC-23C with Pf3-16C the mean time required for the insertion process was analyzed at 15°C, 20°C, 25°C and 30°C. The insertion rates *k* were calculated by the reciprocal of the time required for a distance change from 6.2 to 4.0 nm. As shown in Fig. 5 (B) the insertion rate at 15°C was determined to *k* ≈ 250 s^−1^ and indeed increased to values around 500 s^−1^ at 30°C. Such a behaviour is expected for a catalyzed process and a rate in the range of 500 s^−1^ appears reasonable. The activation energy *E_A_* of the YidC catalyzed insertion process was determined by an Arrhenius analysis of the data. The Arrhenius plot in Fig. 5 (C) was fitted to
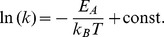



The activation energy was calculated to be *E_A_* = (5.3±2.6) 10^−20^ J per molecule or *E_A_* = (32±16) kJ mol^−1^.

### The Mutant 19A-Pf3-16C does not Interact with YidC

**Figure 6:**
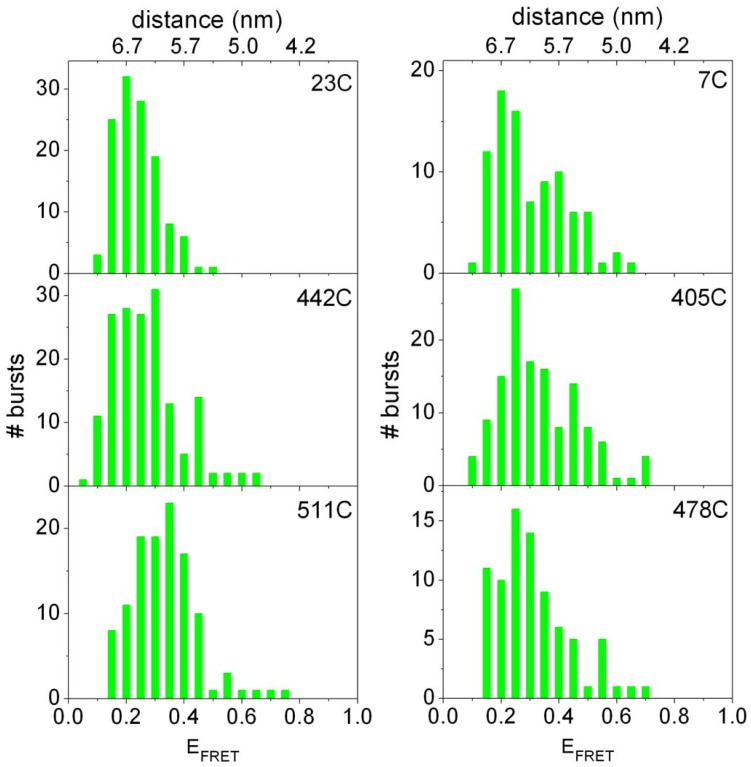
Distribution histograms of photon burst. FRET events between Atto520 labeled Pf3-19A16C coat and Atto647N labeled YidC-23C, YidC-442C, YidC-511C, YidC-7C, YidC-405C and YidC-478C were recorded, respectively.251659264

A Pf3 coat protein mutant with a stretch of 19 Ala residues replacing the transmembrane segment has been characterized to be deficient for membrane insertion *in vivo* [15]. To analyze its interaction with YidC a cysteine residue was introduced at position 16 (19A-Pf3-16C). The mutant was purified and labeled with Atto520 for single-pair FRET. This mutant showed only low FRET efficiencies for all YidC mutants (Fig. 6) with broader distributions in the case of cytoplasmically labeled YidC than for periplasmically labeled YidC proteins. The periplasmic labels could not be contacted by the mutant Pf3 coat protein and the minimal distance was limited to 6 nm (left panels) indicating that the N-terminal region of 19A-Pf3-16C is blocked for translocation. The cytoplasmic labels at the YidC mutants were also limited to be approached by the 19A-Pf3-16C to 5 nm (right panels) indicating that the direct binding of 19A-Pf3-16C to YidC is inhibited. This is in accordance with the observation of intrinsic tryptophan fluorescence that the binding of 19A-Pf3 to YidC is greatly disturbed [16]. In conclusion, the specific binding of the Pf3 protein to YidC clearly depends on hydrophobic interactions.

## Discussion

**Figure 7:**
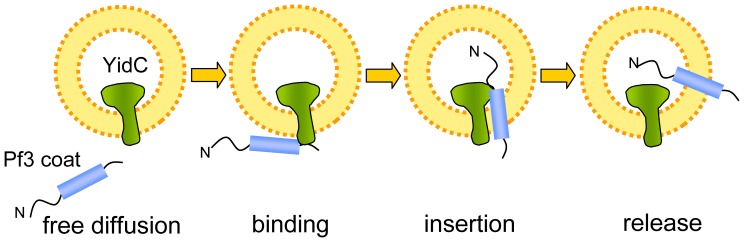
Steps of YidC-assisted protein insertion into the membrane. The Pf3 coat protein binds at the cytoplasmic face to YidC, the N-terminal region becomes translocated and the protein is released from YidC into the lipid bilayer.

This study shows that membrane translocation of the N-terminal domain of the Pf3 coat protein occurs within milliseconds resulting in a close transient contact with all 3 periplasmic loops of YidC. All traces of YidC-bound Pf3 protein showed high FRET efficiencies for only a very short time (Fig. 4). This suggests that after membrane insertion the Pf3 protein is readily released from YidC and then looses its contact to both the periplasmic and the cytoplasmic sites of YidC for its final integration into the lipid bilayer (Fig. 7). These events have been postulated earlier [17] but the time-resolved separation of YidC and its substrate has not been documented so far. The single-pair FRET analysis has shown that binding, insertion and release into the bilayer of a single membrane-spanning protein takes in total about 20 ms (Figs. 4, 5). During this time the hydrophilic N-terminal domain of the protein has to be translocated from the cytoplasmic to a periplasmic location. Presently, it is unknown how this step is achieved. One possibilty is that the hydrophilic domain is shielded by YidC from contacting the lipids during its translocation. It has been shown that size and charge of this region have a strong influence whether it can be translocated by YidC [18]. Previously, we have shown that the orientation of the Pf3 coat protein is strictly determined by the charged residues present in the two hydrophilic regions; in the wild type the N-terminus is always in the periplasm [19]. Also in the reconstituted system, only one orientation is found when the Pf3 protein is labeled with an Atto-dye. When the label was placed at the N-terminal domain of Pf3 it was resistant to externally added quencher showing that it was in the lumen of the proteoliposome [11]. Membrane insertion of the Pf3 coat protein requires the presence of YidC since the N-terminal label was not translocated into YidC-free liposomes [11]. In general, YidC accepts only small domains for translocation, the longest being the periplasmic loop of 29 residues in the mechanosensitive channel protein MscL [20]. How are these hydrophilic regions crossing the membrane bilayer? A possible mechanism is that hydrophobic interactions drive the membrane insertion. When the hydrophobic region of the Pf3 coat protein is bound strongly by the transmembrane segments of YidC the hydrophilic region might drag the N-terminal hydrophilic region across the bilayer to achieve translocation. Hydrophobic contacts have been found between the Pf3 substrate and YidC across the entire transmembrane segments [9].

The time-resolved FRET analysis allowed us to determine the insertion rate for a single YidC molecule, i.e. the time the YidC needs for the translocation of the N-terminal hydrophilic region of a single Pf3 coat protein from the outside into the lumen of the membrane vesicle. The calculated insertion rate is 500 s^−1^ at 30°C.

A calculation of the energy needed to translocate the 18 amino acid residue long hydrophilic N-terminal region of Pf3 coat protein into the lumen of the vesicle, based on the GES scale [21] considering the translocation of the peptide bonds [22], gives a value of 56 kJ mol^−1^ [19]. When the mutation at position 16 to a cysteine is taken into account this value changes to 31 kJ mol^−1^. Compared to this theoretical value, the calculated activation energy of *E_A_* = (32±16) kJ mol^−1^ seems plausible, showing the catalytic activity of YidC as a membrane insertase.

## Materials and Methods

### Generation, Expression and Purification of YidC Mutants

YidC single cysteine mutants (all with a C-terminal His_10_ tag) were obtained after site directed mutagenesis [19] where the natural Cys at position 423 had been changed to Ser (cysteine-less background). A single amino acid was substituted to Cys at the indicated positions, respectively. The mutants were subcloned into the expression vector pMS119EH or pGZ119EH [23] and transformed into *E. coli* C43 cells. The cultures were grown to exponential phase and harvested. The cells were disrupted by French press at 8,000 psi in two passages after the addition of a Complete® protease inhibitor cocktail tablet (Roche) and 5 mM MgCl_2_ and Benzonase® (Merck, 250 U/µl) according to manufacturer instruction. Cell debris was separated from crude lysate at 6,000×g at 4°C and the membrane fraction was collected by centrifugation for 50 min at 150,000×g at 4°C. The membranes were solubilized on ice for 1 h with 1% (w/v) dodecyl maltoside in buffer A [20 mM Tris-HCl (pH 7.5), 150 mM NaCl, 15 mM imidazol, 0.4 mM Tris-(2-carboxyethyl)phosphine (TCEP, Invitrogen)]. After centrifugation for 1 h at 150,000×g at 4°C to remove all non-solubilized components the supernatant was incubated with a Ni^2+^-NTA matrix (Qiagen) for 1.5 h at 4°C and washed with buffer B [20 mM Tris-HCl (pH 7.5), 150 mM NaCl, 40 mM imidazol, 0.4 mM TCEP and 0.05% (w/v) DDM]. YidC was eluted in 2 mL fractions with buffer C [20 mM Tris-HCl (pH 7.0), 100 mM NaCl, 400 mM imidazol, 0.4 mM TCEP] containing 0.05% (w/v) DDM.

### Complementation of YidC Mutants

The plasmids encoding the single-cysteine mutants of YidC were transformed into the YidC depletion strain MK6S. In this strain, the chromosomal *yidC* gene is under control of the arabinose BAD promoter [10]. Cells were grown overnight in LB medium containing 0.2% (w/v) arabinose. Depletion of the chromosomally-encoded YidC was observed after growth in the presence of 0.2% (w/v) glucose for 3 h at 37°C. The cells were diluted in LB medium and 3 µl of each dilution were spotted on LB agar plates containing 0.2% (w/v) glucose or 0.2% (w/v) arabinose, respectively, and, when appropriate, 0.2 mM IPTG. The plates were incubated overnight at 37°C. As a control, cultures with the empty vectors and a culture expressing the wild-type YidC were spotted.

### Expression and Purification of Pf3 Coat Protein Mutants

The single cysteine mutants Pf3-16C, Pf3-48C and Pf3-19A16C were expressed from *E. coli* C43 cells. Pf3-19A16C is a mutant where the membrane-spanning region (residues 19–34) was replaced by 19 Ala residues and the Gln at position 16 was replaced by a Cys. Cells (5 g) were resuspended in 25 mL 100 mM Tris-HCl buffer (pH 8.0) and disrupted by French Press at 8000 psi. Cell debris was sedimented by centrifugation for 10 min at 5,000×g at 4°C followed by ultracentrifugation for 1 h at 150,000×g at 4°C to sediment the membrane fraction. The membrane was solubilized in 5 mL 100 mM Tris-HCl (pH 8.0) buffer containing 8 M urea for 2 h at 18°C. The solubilized membrane proteins were fractionated by reversed phase chromatography followed by size exclusion chromatography using a Superdex 200 prep grade column with 100 mM Tris-HCl (pH 7.5) buffer containing 10% (v/v) isopropanol followed by a Superdex 75 prep grade column (Amersham Pharmacia) with 100 mM Tris-HCl (pH 7.0) buffer containing 10% (v/v) isopropanol.

### Fluorescence Labeling of YidC and Pf3 Coat Mutants

Solubilized YidC was mixed with equimolar amounts of Atto647N maleimide (Atto-Tec), dissolved in DMSO, and incubated on ice for at least 30 min. Purified Pf3 coat was mixed with 5-fold molar excess of TCEP and incubated with equimolar amounts of Atto520 maleimide (Atto-Tec), dissolved in DMSO, for at least 30 min at 20°C. Unconjugated dye was separated from both samples by size exclusion chromatography using a Superdex 200 prep grade column (GE Healthcare). The elution buffer for YidC protein was 20 mM Tris-HCl (pH 7.0), containing 100 mM NaCl and 0.03% (w/v) DDM, and for Pf3 coat protein 100 mM Tris-HCl (pH 7.0) containing 10% (v/v) isopropanol. Before use both buffers had been treated with charcoal in the absence of DDM and isopropanol, respectively, to absorb spectroscopically disturbing contaminants. In order to remove small charcoal particles the solutions were filtered using a sterile syringe filter (cellulose acetat, 0.2 µm pore size, Whatman).

The purities and the labeling efficiency of the proteins were controlled using sodium dodecyl sulfate−polyacrylamide gel electrophoresis (SDS−PAGE) together with fluorescence gel imaging (Typhoon Trio+, GE Healthcare). The gel was excited with 532 nm and emission of the Atto520 dye was detected in a range between 545 and 565 nm. For detecting Atto647N, the gel was excited with 635 nm and emission was detected in the range between 655 and 685 nm with a mean sensitivity of the photomultiplier. The homogeneity of the labeled proteins was tested by their fluorescence autocorrelation and anisotropy (Text S4 and S5).

### Preparation of Liposomes and Proteoliposomes

1,2-Dioleoyl-sn-glycero-3-phosphocholine (DOPC) were purchased from Avanti Polar Lipids (Alabaster, AL) and prepared as described [24]. The dry lipid film was resuspended in 20 mM Tris-HCl buffer (pH 7.0) containing 100 mM NaCl, frozen in liquid nitrogen, and then stored at –80°C. Phosphate of the DOPC sample was determined according to Ames and Dubin [25]. Liposomes were generated by extrusion technique (Mini-Extruder, Avanti Polar Lipids Inc). 200 µL of a 700 µM lipid suspension was extruded 17 times through a membrane with a pore size of 0.4 µm resulting in liposomes with a mean particle size of 250 nm as determined by dynamic light scattering (W130i, AvidNano). For preparing proteoliposomes, labeled YidC protein was added to a final concentration of 5 to 10 nM and extruded with the lipids as described above.

### Confocal FRET Microscopy

A 20 µL droplet containing an adequate dilution of proteoliposomes (in 20 mM Tris-HCl buffer (pH 7.0), 100 mM NaCl) was applied onto a cover slip. This ensured that only single Atto647N-labeled YidC proteoliposomes were diffusing through the confocal volume. To this droplet, 20 µL of a diluted solution of the Atto520-labeled Pf3 coat protein (in 20 mM Tris-HCl buffer (pH 7.0), 100 mM NaCl, 5% (v/v) isopropanol) was added. Fluorescent signals were recorded between 530 to 590 nm (FRET donor), and between 663 to 737 nm (FRET acceptor) using two synchronized TCSPC cards. Alternating laser excitation (ALEX) allowed to excite the FRET donor fluorescence at 514 nm and the acceptor fluorescence at 635 nm, respectively [26].

Time traces of individual fluorescent bursts were followed (Text S1) and recorded for 420 s. The bursts events were individually analysed (Text S3) for FRET efficiencies and donor – acceptor distances using custom-designed computer programs as described [27].

## Supporting Information

Text S1 **Single molecule data analysis.**
(DOC)

Text S2 **Confocal Setup with Alternating Laser Excitation.**
(DOC)

Text S3 **FRET histograms.**
(DOC)

Text S4 **Fluorescence autocorrelation.**
(DOC)

Text S5 **Anisotropy.**
(DOC)
