# Supplementary material for: Real Time Observation of Single Membrane Protein Insertion Events by the Escherichia coli Insertase YidC
Source: PLoS One. 2013 Mar 19;8(3):e59023. doi: 10.1371/journal.pone.0059023 (PMC3602594; doi:10.1371/journal.pone.0059023)
Supplement: Text S1 — Single molecule data analysis. (DOC) [file pone.0059023.s001.doc]

**Supporting Information to: Winterfeld et al.**

## **S1. Single molecule data analysis**

The fluorescence intensities of both detecting channels were adjusted with solutions of donor and acceptor dye only for the single-molecule measurements. For background correction the fluorescence intensity of the donor and acceptor channels with buffer solution was measured. Briefly, for the FRET donor channel, a background of 5-8 counts/ms was subtracted. Also, the detection efficiencies *η* of both detection channels as well as the quantum yields *Φ *of both dyes (Atto520: *Φ_D_* =90%, *η_D_*= 0.33; Atto647N: *Φ_A_* = 65%, *η_A_* = 0.39) were taken into account. With these values the correction factor

$\gamma=\frac{\varphi_{A} \eta_{A}}{\varphi_{D} \eta_{D}}$ (1)

was determined to γ = * *0.85.

| YidC mutant +  Pf3-16C coat | number of bursts | number of bursts with FRET events | number of bursts with FRET events and change in E_FRET_ |
| --- | --- | --- | --- |
| 7C | 1930 | 79 (4.1%) | 17 (21.5%) |
| 23C | 8787 | 82 (0.9%) | 54 (65.9%) |
| 405C | 1743 | 145 (8.3%) | 35 (24.1%) |
| 442C | 3627 | 127 (3.5%) | 55 (43.3%) |
| 478C | 1289 | 209 (16.2%) | 25 (12.0%) |
| 511C | 830 | 60 (7.2%) | 28 (46.7%) |

The single-molecule FRET experiments were performed with alternating laser excitation (ALEX) (1, Fig. S1). With this ALEX the excitation was divided into two time windows. In the first time window with a period of 34 ns, the FRET donor was excited with a 514 nm cw-laser line. In the second time window with a period of 16 ns, the acceptor was excited with a 80 ps laser pulse at 635 nm with a pulse rate of 20 MHz. This second excitation is the so called "acceptor test." With this alternating laser excitation scheme the complete fluorescence decay of the acceptor dye was recorded. This approach allows selecting proteoliposomes with only one labeled YidC and to filter out those with more than one labeled YidC or to eliminate photon bursts originating from donor-only events in the absence of acceptor. By gating the microtimes of the detection electronic, the FRET signal was separated from the acceptor-test signal. Time trajectories of the fluorescence signals were further analyzed with the custom software "Burst-analyzer" [2]. This software is a tool to edit the data recorded with the two synchronized TCSPC cards simultaneously. The "Burst-analyzer" divided each 420 s long trace into sections of 30 s and automatically performs a criteria-based search of photon bursts in all sections. Criteria for this search were burst lengths between 14 and 60 ms and count levels between 10 and 100 counts/ms for the donor and FRET signal, respectively. The numbers of bursts, fulfilling these criteria are listed in Table S1 and Table S2, for each experimental condition. In the experiment, most of the detected bursts occurred shortly after the addition of the protein, during the first 4 min. This underlines that they are not the products of simple collision events of two membrane proteins but truly reflect membrane translocation.

**Table S1** Total number of bursts and number of bursts used for the FRET analysis for the experiments with N terminally labeled Pf3-16C coat protein. The numbers in brackets denote the corresponding percentages.

| YidC mutant +  Pf3-48C coat | number of bursts | number of bursts with FRET events | number of bursts with FRET events and change in E_FRET_ |
| --- | --- | --- | --- |
| 7C | 3689 | 107 (2.9%) | 53 (49.5%) |
| 23C | 8569 | 58 (0.7%) | 32 (55.2%) |
| 405C | 2274 | 202 (8.9%) | 84 (41.6%) |
| 442C | 2349 | 91 (3.9%) | 28 (30.8%) |
| 478C | 1515 | 132 (8.7%) | 53 (40.2%) |
| 511C | 914 | 76 (8.3%) | 20 (26.3%) |

**Table S2** Total number of bursts and number of burst used for the FRET analysis for the experiments with C terminally labeled Pf3-48C coat protein. The numbers in brackets denote the corresponding percentages.

In order to calculate the FRET efficiencies as well as the distances the donor and acceptor intensities, $I_{D}$ and $I_{A}$, respectively, were background- and crosstalk-corrected. The FRET efficiency was calculated with

$E_{FRET}=\frac{I_{A}}{ I_{A}+\gamma\cdot I_{D}}$ (2)

with the calibration factor γ (see eq. 1).

which allows for the different quantum yields of the fluorophores, $\varphi_{D}$ and $\varphi_{A}$, and their different detection efficiencies, $\eta_{D}$ and $\eta_{A}$. The absolute, time-dependent donor-acceptor distance *d* is calculated; using the Förster radius *R_0_*, by

$d=R_{0}\left(\frac{1}{E_{FRET}}-1\right)^{\frac{1}{2}}$ (3)

The Förster radius for FRET pair Atto520 / Atto647N was calculated to 5.3 nm (Atto-Tec). Fluorescence correlation spectroscopy (FCS) was used to inspect the homogeneity of the Pf3 coat samples as well as the proteoliposomes preparations.

FCS data were analyzed by fitting the ACFs to

$G(\tau)=\frac{1}{N_{F}}\left(\frac{1}{1+\tau/\tau_{D}}\right)\left(\frac{1}{1+\left(\tau/\tau_{D}\right)\left(\omega_{ 0}/z_{ 0}\right)^{2}}\right)^{\frac{1}{2}}$ (4)

where *N_F_* is the average number of fluorescent molecules in the Gaussian shaped detection volume with the 1/e^2^ radii, *ω_0_* and *z_0_*, in radial and axial direction, respectively, and *τ_D_* is the translational diffusion time of the observed molecules. When the Atto520 labeled Pf3 coat protein was measured in the presence of proteoliposomes the ACF was fitted to

$\begin{matrix} G(\tau)=\frac{1}{N_{F}}\left(\left(C _{1}\left(\frac{1}{1+\tau/\tau_{1}}\right) \left(\frac{1}{1+\left(\tau/\tau_{ 1}\right)\left(\omega_{ 0}/z_{ 0}\right)^{2}}\right)^{\frac{1}{2}}\right)+\right. \\ \left.(1-C _{1})\left(\frac{1}{1+\tau/\tau_{2}}\right) \left(\frac{1}{1+\left(\tau/\tau_{2}\right)\left(\omega_{ 0}/z_{ 0}\right)^{2}}\right)^{\frac{1}{2}}\right) \end{matrix}$ (5)

where *C_1_* is the ratio of bound to unbound protein, *τ_1_* and *τ_2_* are the translational diffusion times of both populations. The diffusion constant *D* was calculated with $D=\omega_{0}^{2}/4\tau_{D}$whereas the optical parameters ω_0_  and (ω_0_/z_0_)^2^ were determined by fitting the ACF of a rhodamine110 solution (*D* = 2,8 x 10^-6^ cm^2^ / s; 4) to eq.3.

***References***

1. Zarrabi N, Heitkamp T, Greie JC, Börsch M (2008) Monitoring the conformational dynamics of a single potassium transporter by ALEX-FRET. *Proc SPIE* 6862**:**68620M.

2. Zarrabi N, Ernst S,Düser MG,Golovina-Leiker A, Becker W et al (2009) Simultaneous monitoring of the two coupled motors of a single FoF1-ATP synthase by three-color FRET using duty cycle optimized triple-ALEX. *Proc SPIE* **7185:**18505.
