# Supplementary material for: Real Time Observation of Single Membrane Protein Insertion Events by the Escherichia coli Insertase YidC
Source: PLoS One. 2013 Mar 19;8(3):e59023. doi: 10.1371/journal.pone.0059023 (PMC3602594; doi:10.1371/journal.pone.0059023)
Supplement: Text S2 — Confocal Setup with Alternating Laser Excitation. (DOC) [file pone.0059023.s002.doc]

**Supporting Information to: Winterfeld et al.**

***S2. Confocal Setup with Alternating Laser Excitation***

For the single-molecule measurements a confocal setup was used as described earlier [1]. For the excitation of both FRET fluorophores two lasers were used. Pf3 coat protein labeled with the donor Atto520 was continously excited at 514 nm (Series 2000, Spectra Physics) and attenuated to a power of 100 µW to avoid bleaching of the donor fluorophore. For the acceptor test the YidC protein labeled with the acceptor Atto647N was excited by a 80 ps laser pulse at 635 nm (LDH 635B, PicoQuant) with a repetition rate of 20 MHz (PDL808 Sepia, PicoQuant). The laser was attenuated to a power of 30 µW. Both lasers were triggered and synchronized by a programmable external arbitrary waveform generator (AWG 2041, Tektronix) [2]. In order to obtain an enlarged confocal volume, both laser beams were compressed by lens pairs. For the 514 nm and 635 nm laser lens pairs with focal lengths of 150 mm / 50 mm and 250 mm / 80 mm were used, respectively. Both lasers were manually overlapped at the back aperature of the microscope objective (UplanSApo 60x, N.A. 1.2, Olympus) using a dichroic beam splitter (DCXR 540, AHF Analysentechnik).


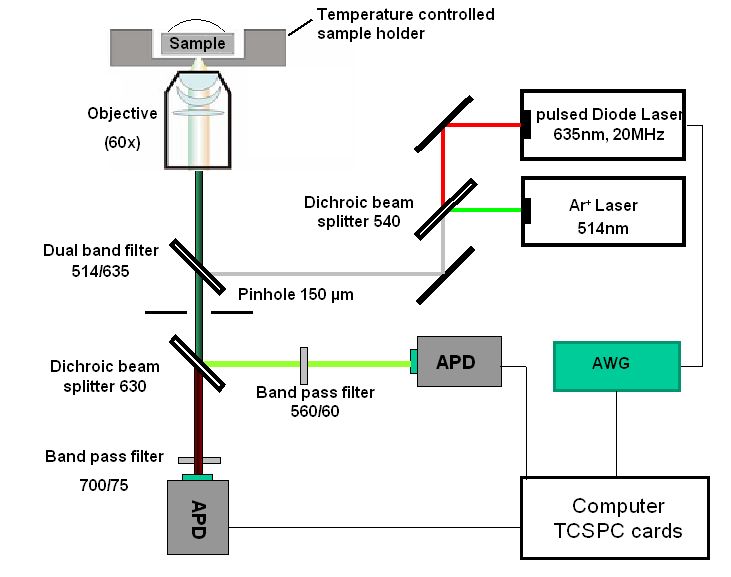


**Fig. S1** Experimental setup, see text for abbreviations.

After entering the epifluorescent port of the microscope the laser beams were redirected by a dichroic filter (514/635, AHF Analysentechnik) and focussed by the objective into a droplet of 40 µl. For the measurements 20 µL of proteoliposomes and 20 µL of Pf3 coat protein, diluted in 20 mM Tris-HCl buffer (pH 7.0) containing 100 mM NaCl and 5% (v/v) isopropanol to a concentration of single molecule level, were mixed directly onto the cover slip and the measurement over 420 s was started immediately. The fluorescence emission signal was separated from parasitic excitation laser light with a dual line beamsplitter (dual band 514/635, AHF), and was separated, after passing a 150 μm pinhole, into two different spectral channels with a dichroic beam splitter (DCXR 630, AHF Analysentechnik). The fluorescent signal of Atto520 was detected between 530 and 590 nm and the signal of Atto647N between 663 nm and 737 nm using band pass filters (HQ 560/60 for Atto520, HQ 700/70, AHF for Atto647N, both Filters from AHF Analysentechnik) mounted in front of the avalanche photodiodes (APD, SPCM-AQR-14, Perkin Elmer). Photons were counted using two synchronized TCSPC cards (SPC 152, Becker & Hickl, Germany). The setup is shown in Fig. S1.

***References***

1. Pristinski D, Kozlovskaya V, Sukhishvili SA (2005) Fluorescence correlation spectroscopy studies of diffusion of a weak polyelectrolyte in aqueous solutions. *J Chem Phys* 122:14907-14915.

2. Zarrabi N, Ernst S,Düser MG et al (2009) Simultaneous monitoring of the two coupled motors of a single FoF1-ATP synthase by three-color FRET using duty cycle optimized triple-ALEX. *Proc SPIE* **7185:**18505.
