# Supplementary material for: Real Time Observation of Single Membrane Protein Insertion Events by the Escherichia coli Insertase YidC
Source: PLoS One. 2013 Mar 19;8(3):e59023. doi: 10.1371/journal.pone.0059023 (PMC3602594; doi:10.1371/journal.pone.0059023)
Supplement: Text S3 — FRET histograms. (DOC) [file pone.0059023.s003.doc]

**Supporting Information to: Winterfeld et al.**

***S3. FRET Histograms***

All photon bursts of periplasmically labeled YidC mutants (23C, 442C, 511C) as well as cytoplasmically labeled YidC mutants (7C, 405C, 478C) in the presence of Pf3-16C or Pf3-48C coat protein, respectively, were analyzed as described in *Material and Methods*. The distributions of maximum FRET efficiencies of each experiment are shown in Fig. S2 The results include bursts that do not show alterations of FRET efficiencies and thereby no distance changes, as well as bursts which show alterations of FRET efficiencies and therefore distance changes between the labeled proteins. Periplasmically labeled YidC mutants (left panel) showed narrowed distributions in comparison to the cytoplasmically labeled YidC mutants (right panel). This could be due to the orientation of the labels to each other. In the case of the cytoplasmically labeled YidC proteins the probes of both Pf3 coat mutants (16C and 48C) come in close contact to the YidC label during the insertion process. In contrast, the periplasmically labeled YidC proteins show distinct distributions since initially donor and acceptor are clearly separated by the lipid bilayer.


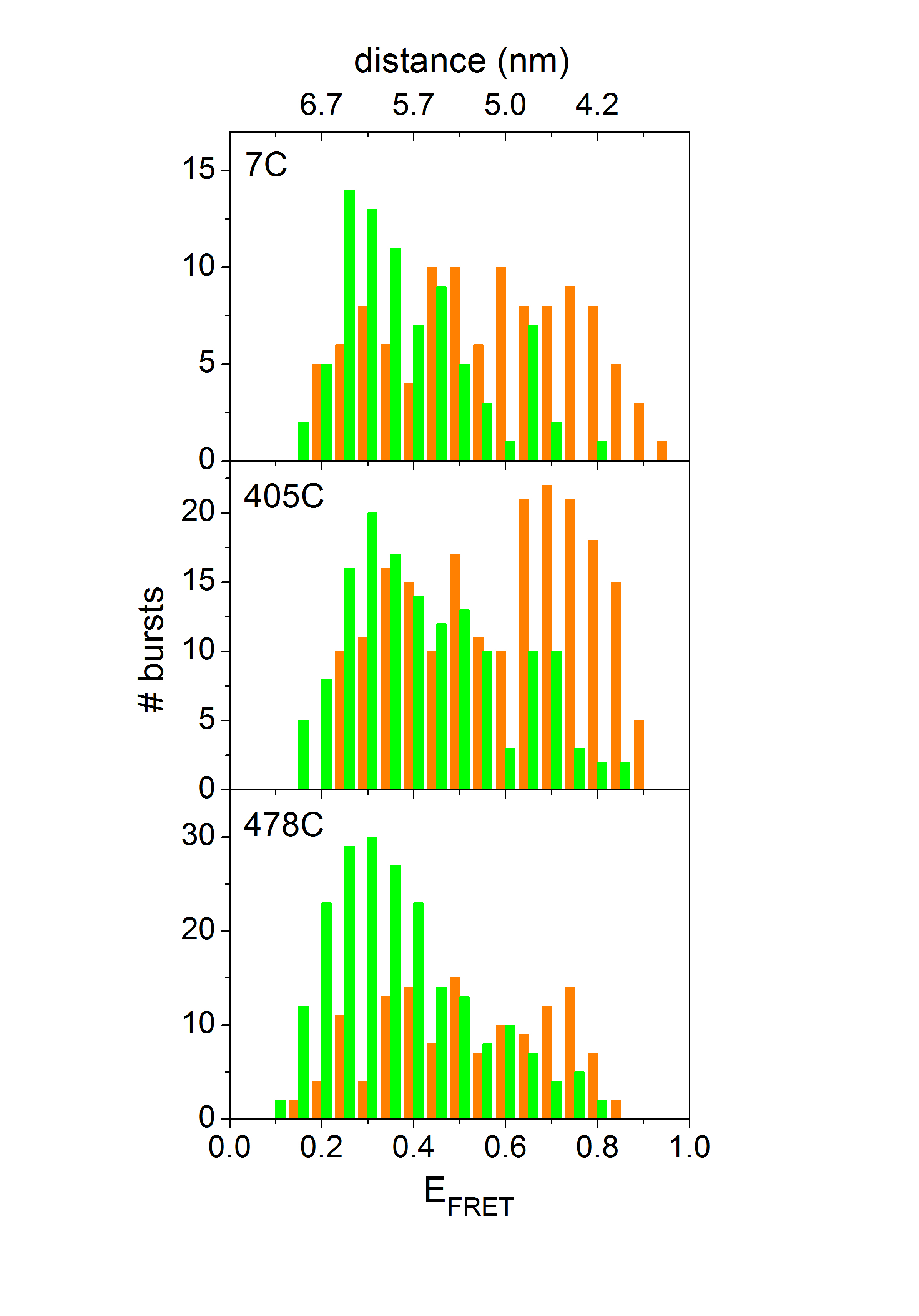

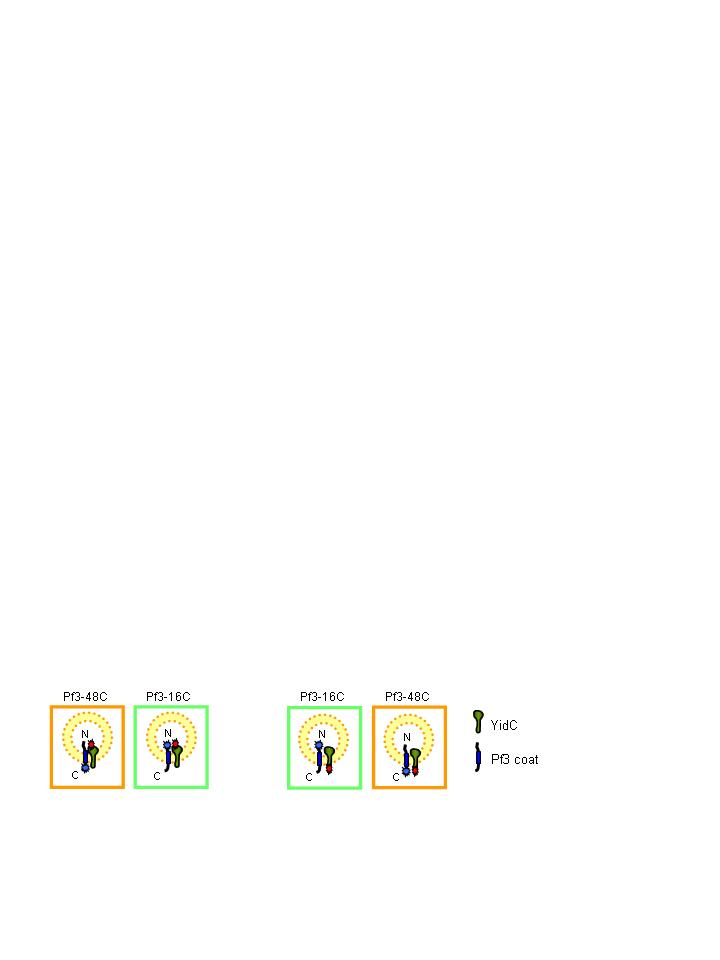

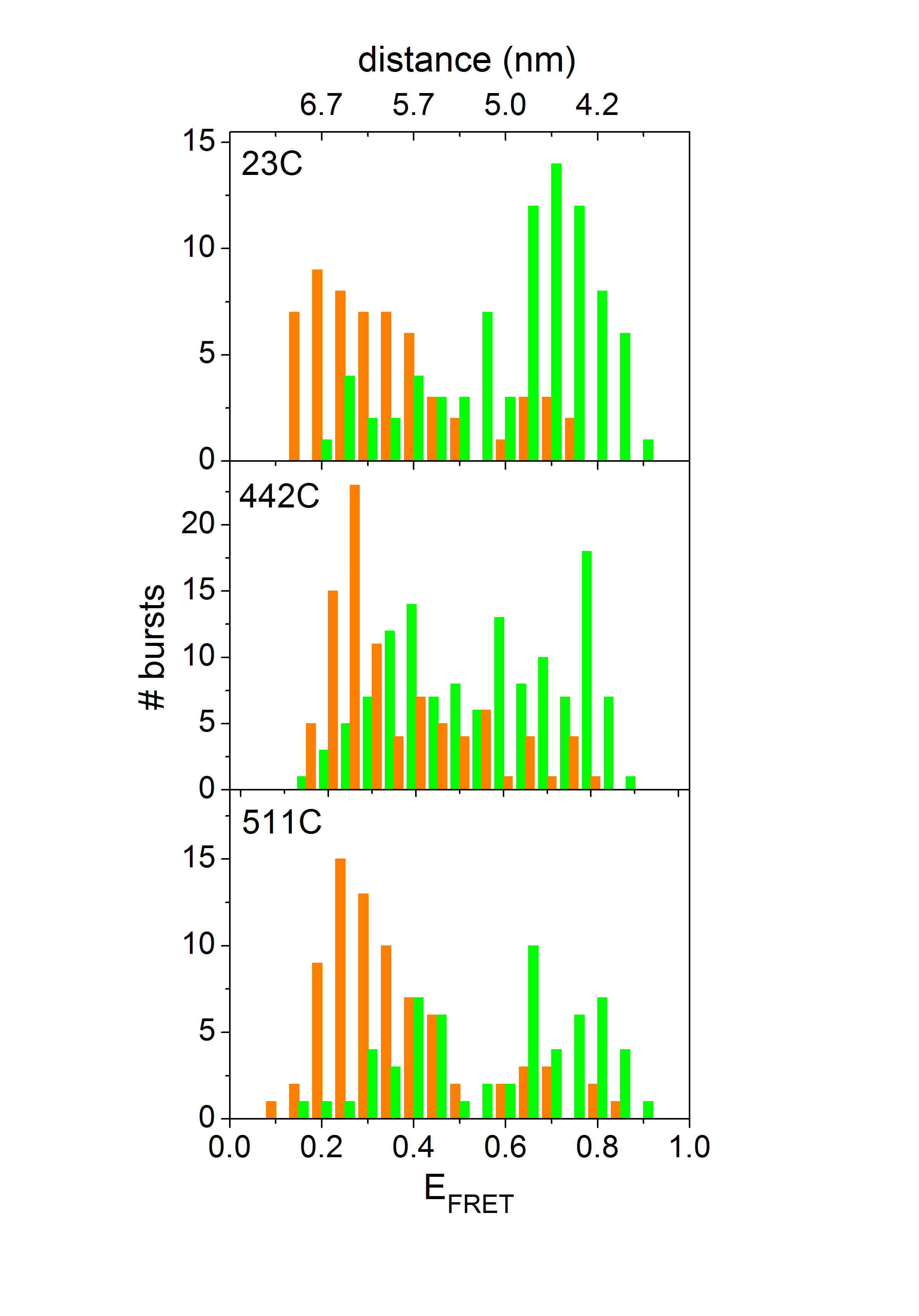


**Fig.S2** Histograms of maximum FRET efficiencies (E_FRET_) of all burst of Atto647N labeled YidC mutants. *Left panel*: histograms for periplasmically labeled YidC in contact with Pf3 coat protein labeled at residue 16C (green bars) or 48C (orange bars), respectively*. Right panel*: histograms of cytoplasmically labeled YidC in contact with Pf3 coat protein labeled at residue 16C (green bars) or 48C (orange bars), respectively*.* The labeled residue within the YidC protein is indicated in each histogram and the donor-acceptor distance calculated from E_FRET_ is displayed on the upper axis.

When only the bursts which show alterations of FRET efficiencies were considered the histograms become more significant (Fig. 2, 3). Periplasmically labeled YidC (left panel) showed high FRET efficiencies only with the N-terminally labeled Pf3-16C (green bars) whereas with the C-terminally labeled Pf3-48C (orange bars) low FRET efficiency values were found. The results show that the N-terminal region of Pf3 coat is in close contact (3 - 4 nm) with the periplasmic domains of the insertase whereas the C terminal region is separated by 6 - 7 nm indicating that the coat protein is in its transmembrane configuration.

From the experiments with cytoplasmically labeled YidC (right panel) high FRET efficiencies were found with Pf3-48C coat (orange bars) suggesting that the C terminal region of the coat protein closely contacts (3 - 4 nm) the cytoplasmic domains of YidC after binding and insertion, respectively. Some high FRET signal were detectable with Pf3-16C which can be explained as events prior the membrane translocation of the probe.
