# Supplementary material for: Real Time Observation of Single Membrane Protein Insertion Events by the Escherichia coli Insertase YidC
Source: PLoS One. 2013 Mar 19;8(3):e59023. doi: 10.1371/journal.pone.0059023 (PMC3602594; doi:10.1371/journal.pone.0059023)
Supplement: Text S4 — Fluorescence autocorrelation. (DOC) [file pone.0059023.s004.doc]

**Supporting Information to: Winterfeld et al.**

***S4. Fluorescence Autocorrelation***

The homogeneity of the purified proteins and the generated proteoliposomes were probed by measuring their fluorescence autocorrelation functions (ACF). Fig. S3 shows the normalized autocorrelation functions of Atto520 labeled Pf3-16C coat protein and DOPC liposomes containing Atto520 labeled YidC. The diffusion coefficients were obtained by fitting the curves to 1.5 and 1.6. The ACF for the Pf3-16C coat sample could be fitted with high accuracy to eq. 4 indicating a homogeneous, monodisperse protein sample. The calculated diffusion coefficient of *D* = (1.2 ± 0.1) x 10^-6^ cm^2^/s corresponds to a hydrodynamical radius of 2.0 nm in agreement with the theoretical value for an ideal alpha-helix with a length of 40 residues [1]. Proteoliposomes containing Atto520 labeled YidC show a diffusion coefficient of *D* = (4.2± 0.3) x 10^-8^ cm^2^/s in agreement with the expected values for spherical particles with diameter in the 200 nm range. In order to study the diffusion behaviour of the membrane inserted coat protein the ACF of Pf3-16C coat in the presence of YidC proteoliposomes was measured and fitted to eq. 5. From The ACF in Fig. S3 two distinct diffusion coefficients corresponding to the two states of the coat protein could be calculated. Whereas the shorter coefficient with *D* = (1.4 ± 0.2) x 10^-6^ cm^2^/s is attributed to free Pf3 coat protein the other coefficient with *D* = (3.8 ± 0.3) x 10^-8^ cm^2^/s matches the value measured for the Atto520 labeled YidC in DOPC liposomes. The ACF of free Atto520 dye which was used to calibrate the set-up is also shown in Fig. S3.


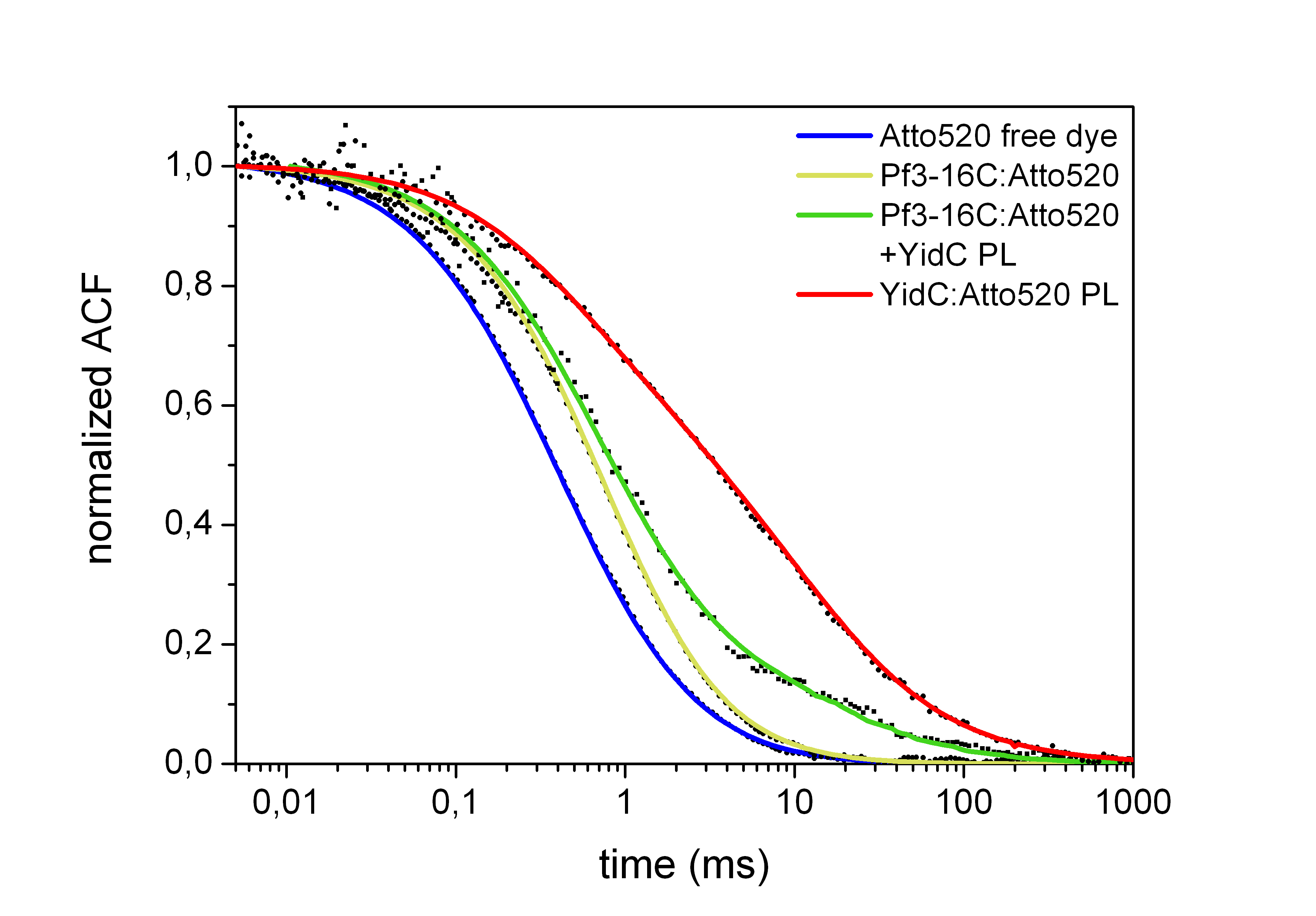


**Fig. S3** Normalized autocorrelation funcions (ACF) of Atto520 labeled Pf3-16C coat, labeled Pf3-16C coat in the presence of YidC proteoliposomes (PL) and proteoliposomes containing Atto520 labeled YidC. For comparison the ACF of the free dye is shown as well.

***Reference***

1. Zagrovic B, Jayachandran G, Millett IS, Doniach S, Pande VS (2005) How large is an alpha-helix? Studies of the radii of gyration of helical peptides by small-angle X-ray scattering and molecular dynamics. *J Mol Biol* 353**:**232-241.
