# Supplementary material for: Real Time Observation of Single Membrane Protein Insertion Events by the Escherichia coli Insertase YidC
Source: PLoS One. 2013 Mar 19;8(3):e59023. doi: 10.1371/journal.pone.0059023 (PMC3602594; doi:10.1371/journal.pone.0059023)
Supplement: Text S5 — Anisotropy. (DOC) [file pone.0059023.s005.doc]

**Supporting Information to: Winterfeld et al.**

***S5. Anisotropy***

Fluorescence anisotropies of single Pf3 coat proteins (linear polarized excitation with 514 nm) or YidC labeled with Atto647N (linear polarized excitation with 635 nm) reconstituted in DOPC liposomes were measured separately in the confocal microscope using a polarizing beamsplitter in the fluorescence pathway. Fluorescence anisotropy values were calculated for each burst after correction for the APD detection efficiencies. Aqueous solutions of rhodamine 110, erythrosine blue, Atto520 maleimide reduced with DTT and Atto647N in water were used as anisotropy reference dyes. In Fig. S4, single Atto520 maleimide molecules show a static anisotropy of r =0.02 ± 0.04 as expected for free diffusing Atto dyes. However, Atto520 maleimide at the N terminus of Pf3 coat protein shows an increased anisotropy of r= 0.05 ± 0.06 indicating that the fluorophore is only slightly restricted in motion. The static anisotropy of Atto647N at position 23 of reconstituted YidC was r = 0.11 ± 0.06 indicating a restricted mobility of the label compared to the free diffusing dye. Taking both fluorophore anisotropies into account, the error for the FRET distance measurements can be calculated to be smaller than 20% [1].

**
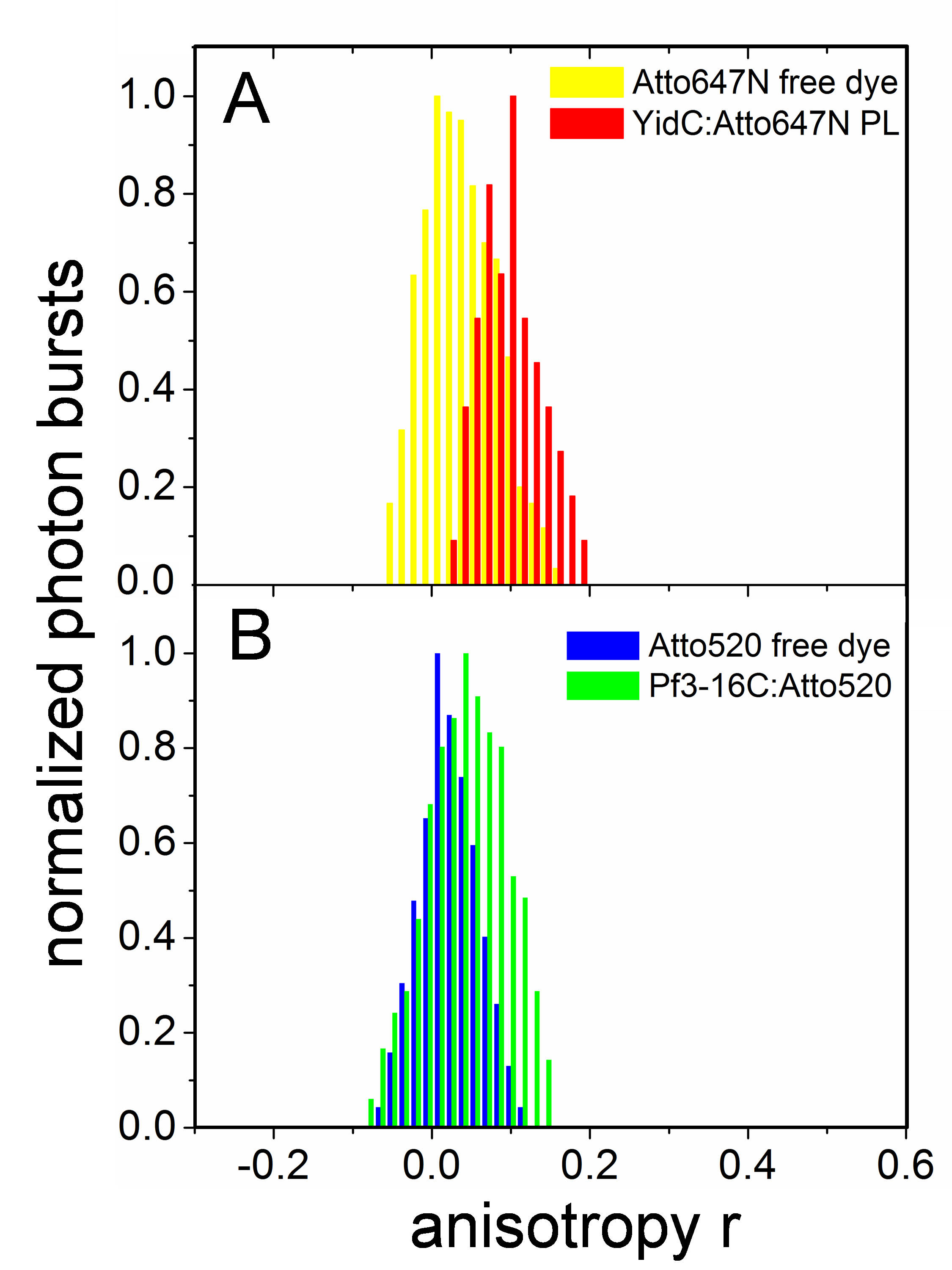
**

**Fig. S4** Static anisotropy distributions for the donor (A) and the acceptor (B) compared with the corresponding free diffusing dye

***Reference***

1. Dale RE, Eisinger J, Blumberg WE (1979) The orientational freedom of molecular probes. The orientation factor in intramolecular energy transfer. *Biophys. J*. 26:161-193.
